# Evaluation of the Chemical, Morphological, Physical, Mechanical, and Biological Properties of Chitosan/Polyvinyl Alcohol Nanofibrous Scaffolds for Potential Use in Oral Tissue Engineering

**DOI:** 10.7759/cureus.29850

**Published:** 2022-10-03

**Authors:** Gamil Al-Madhagy, Ibrahim Alghoraibi, Khaldoun Darwich, Mohammad Y Hajeer

**Affiliations:** 1 Department of Oral and Maxillofacial Surgery, University of Damascus Faculty of Dentistry, Damascus, SYR; 2 Department of Physics, University of Damascus Faculty of Science, Damascus, SYR; 3 Department of Orthodontics, University of Damascus Faculty of Dentistry, Damascus, SYR

**Keywords:** protein adsorption, cytotoxicity, tensile strength test, field emission-scanning electron microscope, atomic force microscope, fourier transformed infrared spectroscopy, natural polymer, biodegradable, biocompatible, chitosan/polyvinyl

## Abstract

Background

Chitosan is a biocompatible, biodegradable, and non-toxic natural polymer that can be fabricated by different methods for use in dental and biomedical fields. Electrospinning can produce polymeric nanofibrous scaffolds and membranes with desirable properties for use in tissue engineering. The objectives of this study were to investigate several morphological, physical, and biological characteristics of these nanofibrous scaffolds and evaluate their potential use in tissue engineering.

Methodology

Chitosan/polyvinyl alcohol nanofibrous scaffolds (CS/PVA NFS) in a ratio of 70/30 were fabricated by conventional electrospinning. The scaffolds were evaluated chemically by Fourier transformed infrared spectroscopy (FTIR) and morphologically by the atomic force microscope (AFM) and the field emission-scanning electron microscope (FE-SEM). These scaffolds were also evaluated mechanically by a tensile strength test and several investigations, including water contact angle, swelling ratio, and degradation ratio. Biological evaluations included protein adsorption, cell culture, and cell viability assay.

Results

The morphological evaluation revealed a homogenous, bead-free mat with an average fiber diameter of 172.7 ± 56.8 nm, an average pore size of 0.54 ± 0.17 µm, and porosity of 74.8% ± 3.3%; the scaffolds showed a tensile strength of 6.67 ± 0.7 Mpa. Scaffolds showed a desired hydrophilic property, as shown by the water contact angle test with a mean angle of 29.5°, while the swelling ratio was 229%, and degradability in phosphate buffer solution after 30 days was 26.9 ± 2.9%. In-vitro cell culture study with adipose tissue mesenchymal stem cells and cell viability and cytotoxicity tests by MTT assay demonstrated well-attached cells with increasing proliferation rate with no signs of cytotoxicity.

Conclusions

Assessment of the CS/PVA NFS revealed randomly oriented bead-free and porous mats. The scaffolds were stable at aqueous solutions following thermal treatment. They were hydrophilic, biodegradable, and biocompatible, as shown by the cell culture and MTT assay, which suggest that the fabricated scaffolds have the potential to be used in tissue engineering applications either as scaffolds, bio-grafts, or barrier membranes.

## Introduction

Polymeric nanomaterials are natural, synthetic, and composite polymers manufactured by one of the nano- approached techniques and have at least one of their three dimensions in the nanoscale [1]. They have attracted great attention lately as many of them are widely available, cheap, easy to process and synthesize, non-toxic, biocompatible, biodegradable, non-antigenic, eco-friendly, and some of them possess intrinsic antimicrobial properties [2,3].

They have been incorporated into various technological applications, such as electrical and electronic industries, water treatment, heavy metal chelating, food industries, and agriculture [4,5], as well as in biomedical fields such as dentistry, pharmacology, and medicine [6,7].

They can be synthesized in various shapes and sizes, such as nanoparticles, nanorods, nanofibers, nanohydrogels, nanosponges, and nanotubes [8,9]. Among these different nanoforms, nanofibers of polymeric materials have been intensively investigated in the biomedical field as they can form scaffolds for tissue engineering, drug carriers, wound dressings, and bio-grafts [10]. The fabrication of these nanofibers can be achieved by various techniques, such as electrospinning, self-assembly, phase separation method, and template synthesis [11].

Electrospinning has been a widely accepted method for fiber fabrication because of its efficiency, simplicity, applicability, and ease of use [12]. During electrospinning, fibers are formed when applying a high-voltage electrostatic field between the needle tip and the collector while the solvent evaporates. The produced nanofibers can form scaffolds with better physical, chemical, and biological properties, such as a high surface area to volume ratio, high tensile strength, high compressibility, good abrasion resistance, high porosity, good biodegradability, and similarity to the extracellular matrix [13].

To fabricate a suitable scaffold for tissue engineering purposes, one should consider using these polymeric nanomaterials for their outstanding properties. Natural polymers such as collagen, silk fibroin, alginate, cellulose, hyaluronic acid, gelatin, elastin, chitin, and chitosan have been studied extensively in this field [14,15].

Chitosan is a unique positively charged biopolymer found in the cell wall of some fungi and yeasts and can be produced by partial or full deacetylation of its ancestor, chitin [16]. In addition to the unique properties of the natural polymeric materials, chitosan has native antibacterial properties [17]; can adhere to negatively charged surfaces; attract cells, proteins, hormones, and growth factors [18]; and has multifunctional active groups that enable it to be modified, interact with, and be cross-linked with a wide variety of materials yielding new composites or enhancing the chemical, physical, and biological properties of chitosan [19]. The excellent biological and chemical properties of chitosan have spotted a light on it as a candidate material for biomedical field applications.

Pure chitosan nanofibers can be produced either by using a high concentration of acetic acid [20] or by using trifluoroacetic acid (TFA) and dichloro methanol (DMO) [21] as solvents, either of which can be environmentally harmful and can have toxicity issues; additionally, the mechanical properties of the produced nanofibers are limited [22]. For these reasons, chitosan nanofibers have been prepared with other natural polymers such as silk fibroin [23], collagen [24], and gelatin [25], or synthetic polymers such as polyvinyl alcohol (PVA) [26], polyethylene oxide (PEO) [27], and polylactic acid (PLA) [28]. These composite nanofibers have better physical, chemical, and biological properties.

PVA is a synthetic polymer that is biocompatible, biodegradable, non-toxic, and electro-spinnable [29]. When blended with chitosan, it causes interference with the rigid connections between chitosan molecules and binds with chitosan by hydrogen bonds and facilitating electrospinning and enhancing the scaffold’s properties [30].

Chitosan has been used in the biomedical field as a hemostatic agent, a wound dressing material, a drug carrier; in the delivery of proteins, growth factors, and vaccines; in anti-tumor therapy; and in biological imaging [31]. It can also be used in soft and hard tissue engineering. It can be used as a bio-scaffold for cell attachment and proliferation, as a grafting material with other polymers and bio-ceramics, or as a barrier membrane in guided bone regeneration and soft-tissue regeneration [32].

In the current work, chitosan/polyvinyl alcohol nanofibrous scaffolds (CS/PVA NFS) were produced by the electrospinning method and then modified by a heat treatment process. There is no previous report that has assessed the chemical, morphological, physical, mechanical, and biological characteristics of these materials. The suitability of these scaffolds in tissue engineering has not yet been investigated. Therefore, the current investigation aimed to assess the chemical composition, morphological structure, tensile strength, wettability, swelling ratio, and degradation rate of these scaffolds. In addition, an assessment of the biological characteristics such as protein adsorption, cell viability, and cell culture was also performed to examine the possibility of implementing these scaffolds in oral tissue engineering.

## Materials and methods

This research work was approved by the University of Damascus Local Research Ethics Committee (ID: DN-30082022-12).

Preparation of solutions

Chitosan powder (medium molecular weight = 190-310 kilodaltons, degree of deacetylation (DDA) = 85%, viscosity 200-800 centipoise), PVA (molecular weight = 78.000 g/mol; hydrolysis 99.8%), glacial acetic acid 99%, absolute ethanol, and phosphate buffer saline (PBS) with a pH of 7.4 (Sigma Aldrich, St. Louis, MO, USA). All chemicals were used without any further purification. Our distillation unit made deionized (DI) water. All the tests were performed at the nanotechnology laboratory, Department of Physics, Faculty of Science, Damascus University.

Chitosan solution was prepared by dissolving 3% weight/volume (W/V) in 2% aqueous acetic acid and stirred using a magnetic stirrer at a speed of 400 rounds per minute (rpm) for 24 hours to obtain a homogenous solution, 10% of PVA was added to 90 mL of DI water and stirred at a speed of 400 rpm for two hours at 60˚C, and then for four hours at room temperature. Both solutions were centrifuged to eliminate undissolved and unsuspended particles, and then both chitosan and PVA were mixed in a ratio of 70/30 under 200 stirring speed for 24 hours at room temperature.

Electrospinning of CS/PVA

The electrospinning apparatus consists of an electrostatic/high-voltage generator (ES813 D50.1, EsdEmc Technology Rolla, MO, USA), a syringe pump (SN 50C6T, Sino device Technology Co, Ltd, China), and a sheet of aluminum as a collector.

CS/PVA solution was loaded onto the syringe and mounted on the pump, the applied voltage was set at a value of 23 kV, the distance between the needle tip and collector was 14 cm, and the pump rate was adjusted at 0.02 mL/hour was kept constant throughout the experiment. The scaffolds were thermally treated at 100˚C for six hours and sterilized by gamma radiation (25 kGy).

Characterization of the produced scaffolds

Chemical Analysis: Fourier Transformed Infrared spectroscopy

Chemical analysis with the Fourier transformed infrared spectroscopy (FTIR) was conducted using a Bruker spectrometer (Bruker Tensor 27 IR, US) with a wavenumber range between 400 and 4,000 and a resolution of 4 cm^-1^. It is used to identify chemical substances and functional groups in different matter forms [33].

Morphological Analysis

Field emission-scanning electron microscope (FE-SEM): Evaluation of the morphological microstructure of the prepared scaffolds was conducted using FE-SEM (MERA3 TESCAN, Brno, Czech). The average diameter of the nanofibers was calculated according to the measurement of 100 randomly selected fibers from different parts of the scaffolds and pore size using ImageJ software (ImageJ, U.S. National Institute of Health; Bethesda, Maryland, USA) [34].

Atomic force microscopy (AFM): Topographical evaluation of the prepared scaffolds was performed using AFM (EasyScan2 FlexAFM, Nanosurf, Leistal, Switzerland) in the tapping mode. Similarly, the average diameter and pore size of the scaffolds were calculated using the device corresponding software program Nanosurf Report Expert v 5.0 (Nanosurf, Leistal, Switzerland).

Mechanical Analysis: Tensile Strength

Tensile strength for heat-treated and untreated CS/PVA mats was performed using a tensiometer (Model M250-2.5CT, Testometric Co Ltd., Lancashire, UK) and using the ASTM D882-12 standard test method [35]. In brief, the nanofibrous mats were cut into small specimens of 10 × 50 mm strips, mounted into the grips with an initial grip distance of 20 mm, and stretched until breakage with a strain rate of 10 mm/minute. Ten samples of each group were measured, and the averages of Young’s modulus, elongation at break, and the ultimate tensile strength were calculated from the stress/strain curve.

Physical Analysis

Water contact angle (WCA): A droplet of deionized water was deposited on the surface of the scaffold, and images were taken at different time intervals (1, 30, and 60 seconds) using a digital camera (Canon PowerShot A520, Canon Inc., NY, USA). The image processing and angle measurements were done to determine the contact angles.

Porosity, pore distribution, and nanofiber density: The percentage of porosity of the prepared scaffolds and the density of nanofibers were determined by the method described by Lim et al. [36]. In brief, absolute ethanol was used as displacement liquid. In brief, the scaffolds were cut into small pieces, and the volume of the scaffolds was taken (Vd). Then the dry weight (W1) was noted and immersed in a known volume of absolute ethanol until saturation. Subsequently, the final weight after immersion was token (W2), and the porosity percentage and the fiber density were calculated using the following equations:

W_eth_ in pores = w2 - w1

Veth = W_eth_ _in pores_/ρV

Porosity %(Ɛ) = {V_eth_/V_d_} × 100.

True volume of fibers V_f_ = V_d_ - V_eth_

The density of the fibers (d) = W_1_/V_f_

Where W_eth in pores_ is the weight of ethanol in pores, V_eth_ is the volume of ethanol in the pores, and ρ is a constant that represents the density of alcohol. The experiment was repeated thrice. The mean and standard deviation values were calculated.

Swelling behavior and degradation rate of the CS/PVA NFS: The swelling ratio of the scaffold was evaluated using the method from Meng et al. [37]. First, the scaffold was cut into five square shapes (about 10 × 10 mm). Then the initial weight of scaffolds (Wi) was noted, and then scaffolds were immersed in distilled water for 24, 48, and 72 hours. In each period, the samples were rinsed, and excessive water was removed by gently dipping them in a filter paper and then weighed in wet condition (Ww). The swelling ratio was calculated according to the following equation:

Swelling ratio % = [(Ww-W_i_)/W_i_] × 100.

The degradation test was conducted by the method described by Agrawal and Pramanik [38]. In brief, the evaluation was done by noting the initial weight of the samples (Wi); the samples were then placed in 5 mL of PBS and incubated at 37°C for 30 days, the samples were taken out from the buffer at specific time intervals and rinsed with deionized water and dried, and their final weight was noted (Wf). The degradation rate was then calculated according to the following formula:

Weight loss % = [(W_i _- W_f_)/W_i_] × 100.

Biological Analysis

Protein adsorption: To determine the amount of protein adsorbed onto the CS/PVA scaffold by ultraviolet-visible (UV-vis) spectroscopy, we used the method described by Liao et al. [39]. First, three samples were soaked in ethanol 70% for one hour and then rinsed with PBS. The samples were then incubated in 10 mL PBS containing 10% fetal bovine serum (FBS) at 37°C incubators for 24, 48, and 72 hours. The samples were then washed with PBS three times to eliminate excess proteins. The washing solution was returned to the FBS solution and quantified using a UV-vis spectrometer. The quantity of protein adsorbed onto the scaffold was calculated by finding the difference between the initial total amount of protein and the non-adsorbed protein.

Cell culture and cell viability assay: Cell culture and cell viability assays were performed using the method described by Ghorbani et al. [40]. Human adipose tissue mesenchymal stem cells (h AD-MSC, Rockville, MD) were trypsinized and cultured in a minimum modified eagle medium (MEM) (Biochrom, Berlin, Germany) containing 10% FBS (Biochrom), 2 mM of L-glutamine (Biochrom), 100 U/mL penicillin (Merck), 100 mg/mL streptomycin, and 1 mM sodium pyruvate (Merck) in a 5% CO_2_ atmosphere at 37˚C incubator. The culture medium was replaced every other day. The fourth passage was used in all cellular experiments. The scaffolds were cut into small rectangular (for cell adhesion and morphology tests) and circular shapes (for cell viability and cytotoxicity test), sterilized by gamma radiation, and put in a culture medium to enhance cell seeding later. For cell morphology and adhesion tests, a certain amount of cell concentration (1 × 10^5^ cells/mL) was seeded on the Cs/PVA scaffolds and tissue culture plate (TCP) as a control sample. The seeded cells were incubated for 72 hours (5% CO_2_ and 37°C), and the medium was changed daily. Scaffolds with cells were prepared for SEM investigation (washed by PBS, fixed, dehydrated, and dried). For the cell viability assay, the tetrazolium salt [3-(4,5-dimethylthiazol-2-yl)-2,5-diphenyltetrazolium bromide] (MTT) was used because the living cells can reduce the MTT tetrazolium compound into a colored formazan product that can be quantified by a spectrophotometer and is directly proportional to the number of living cells in that culture. In brief, the cells were seeded on the CS/PVA mats with culture medium as test groups and on TCPs as a control sample and put in an incubator (5% CO_2_ and 37°C) for 24, 48, and 72 hours separately. The assay was done by aspiring the spent medium and adding 1 mL of MTT solution and 9 mL of fresh medium and incubating for four hours at 37°C and in darkness; absorbance measures were taken using a microplate reader at 570 nm.

## Results

Chemical analysis: FTIR

**Figure 1:**
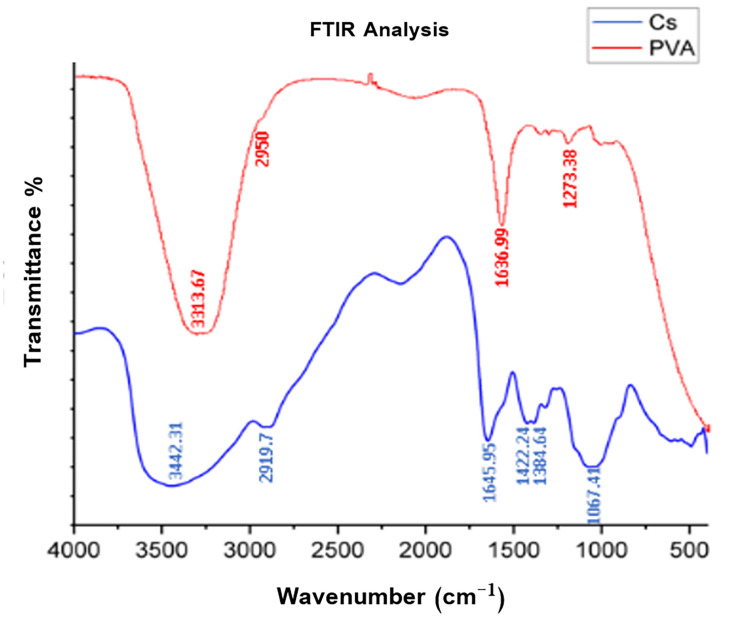
FTIR analysis of pure chitosan (in blue) and polyvinyl alcohol (in red). Cs: chitosan; PVA: polyvinyl alcohol; FTIR: Fourier transformed infrared spectroscopy

The FTIR results of pure chitosan showed a large peak at 3,442 cm^-1^, which refers to O-H and N-H stretching, the 2,919 cm^-1^ peak refers to C-H stretching, while peaks at 1,645 cm^-1^, 1,422 cm^-1^, and 1,384 cm^-1^ represent C=O stretching, N-H bending, and C-O stretching, respectively. The last peak at 1,067 cm^-1^ represents the -C-O-C- glycosidic linkage of chitosan polysaccharide monomers. On the other hand, pure PVA shows typical bands of hydroxyl groups (O-H) at 3,313 cm^-1^ due to O-H stretching, and three peaks at 2,950 cm^-1^, 1,636 cm^-1^, and 1,273 cm^-1^ refer to C-H stretching, C=O and C-O stretching of acetate group, and C-O-H bending, respectively (Figure 1).

**Figure 2:**
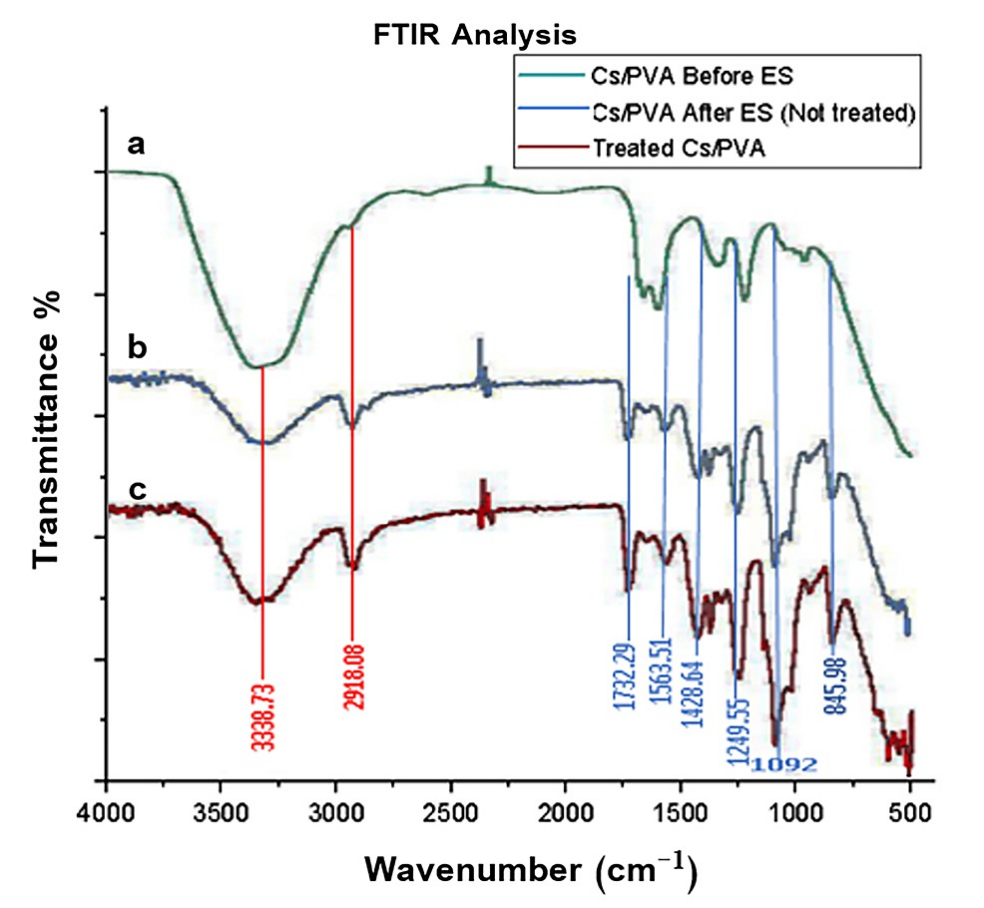
The FTIR analysis of the chitosan/polyvinyl alcohol blend. (A) Before electrospinning. (B) After electrospinning. (C) After heat treatment. Cs/PVA: chitosan/polyvinyl alcohol; ES: electrospinning; Fourier transformed infrared spectroscopy

All characteristic peaks of chitosan and PVA appeared in all spectra with the only difference in the intensity and shape of the peaks or little shifting, as shown in Figure 2.

Morphological analysis

Characterization of the Morphology and Topography of Nanofibers

**Figure 3:**
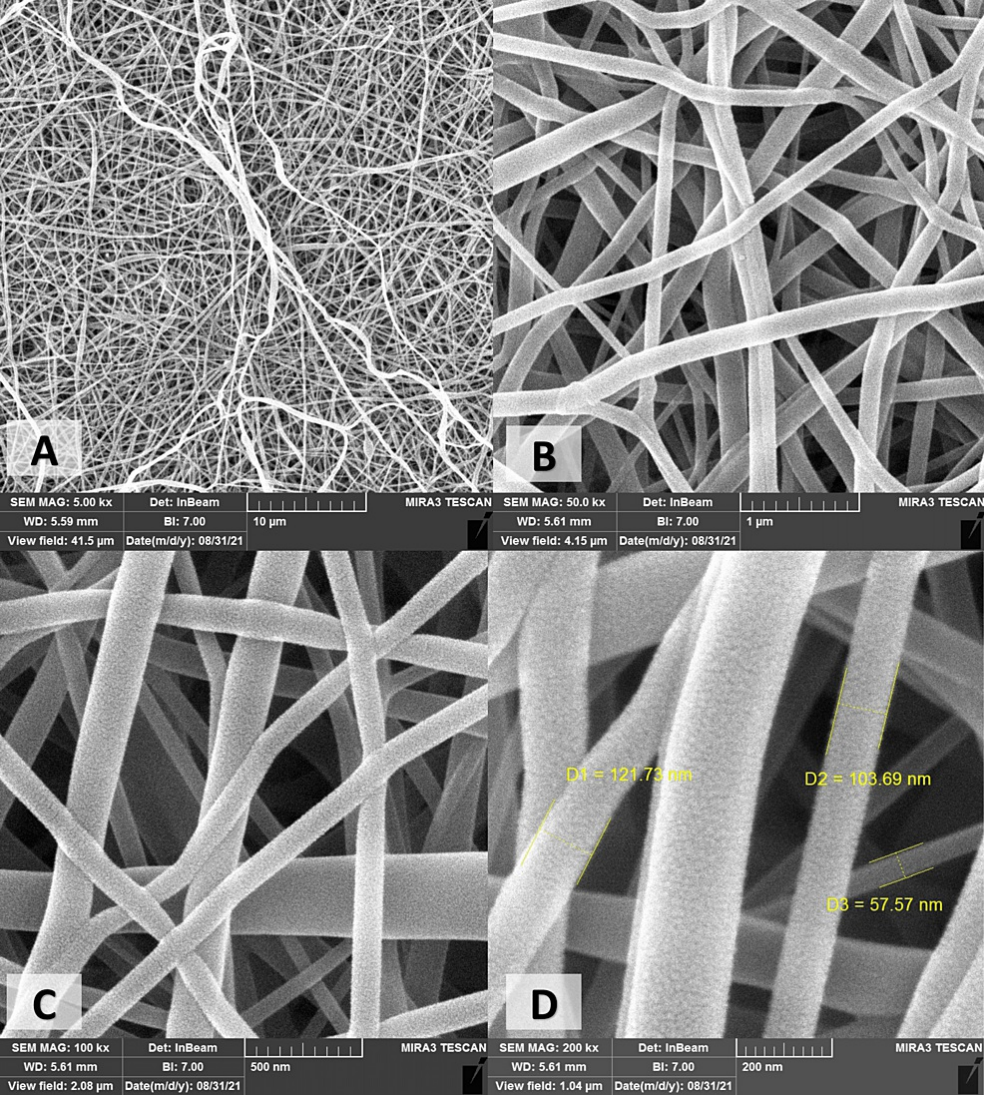
Field emission-scanning electron microscope micrographs of electrospun Cs/PVA obtained in different magnifications: (A) 5,000×. (B) 50,000×. (C) 100,000×. (D) 200,000×. D: diameter of the fiber; Cs/PVA: chitosan/polyvinyl alcohol

**Figure 4:**
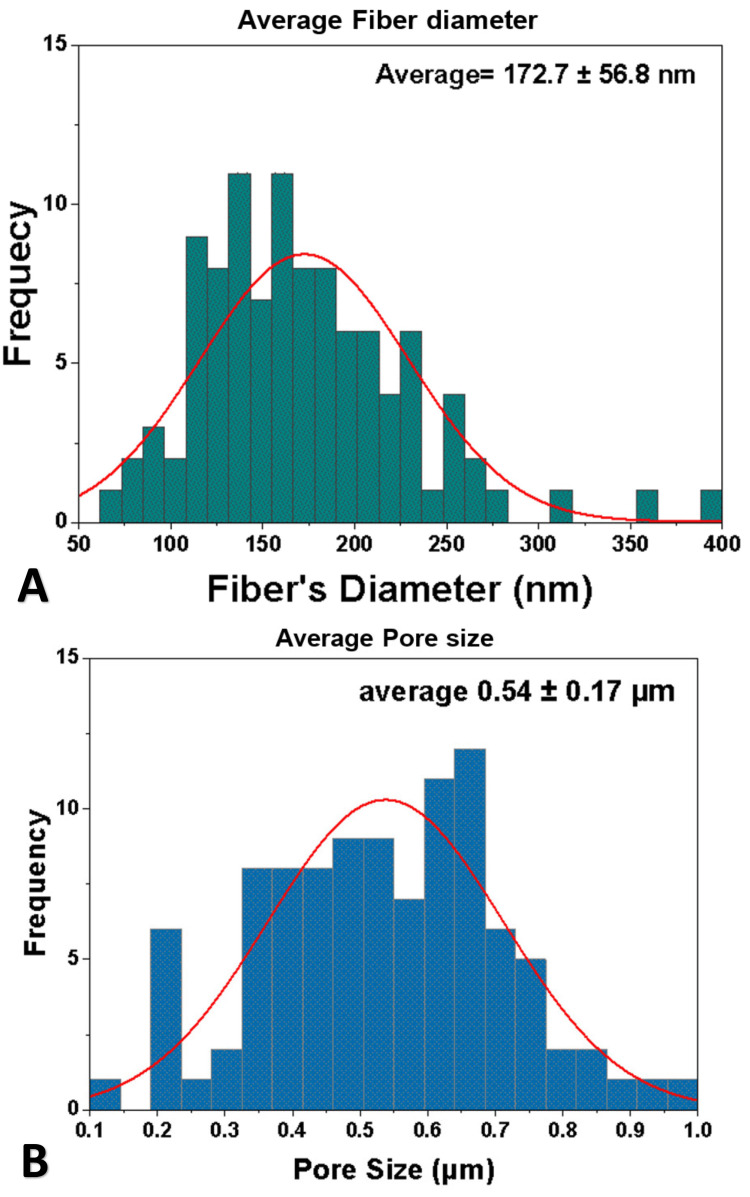
Fiber diameter and pore size of chitosan/polyvinyl alcohol nanofibrous scaffolds. (A) Average fiber diameter. (B) Average pore size.

**Figure 5:**
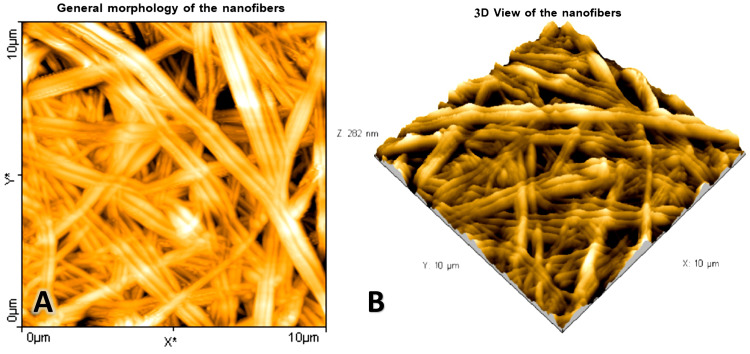
Atomic force microscopy surface micrographs of the chitosan/polyvinyl alcohol nanofibrous scaffolds. (A) General morphology. (B) Three-dimensional view of the scaffold.

The FE-SEM images of the CS/PVA NFS showed continuous, bead-free, and randomly oriented nanofibers with a diameter ranging from 50 to 400 nanometer (nm), with an average diameter of 172.7 ± 56.8 nm (Figures 3, 4), whereas the surface roughness (arithmetical mean height (Sa)) ranged from 68.3 to 101 nm, with a mean Sa of 83.15 ± 10.82 nm, and the root mean square height (Sq) ranged from 86.1 nm to 131 nm, with a mean Sq of 105.3 ± 12.7 nm (Figure 5).

The pore size of the scanned scaffolds ranged from 0.1 to 1 µm, with an average of 0.54 ± 0.17 µm, while the porosity percentage of the test samples was between 69.9% and 79.5%, with a mean value of 74.8% ± 3.3. Fiber density was 0.676 ± 0.058 g/cm^3^, as shown in Table 1.

**Table 1:** Porosity percentage and fiber density of chitosan/polyvinyl alcohol nanofibrous scaffolds.

	Porosity %	Mean ± standard deviation	Density	Mean ± standard deviation
First sample	69.9	74.8 ± 3.3%	0.638 g/cm^3^	0.67 ± 0.058 g/cm^3^
Second sample	75.03		0.63 g/cm^3^	
Third sample	79.48		0.763 g/cm^3^	

Mechanical analysis: tensile strength

**Figure 6:**
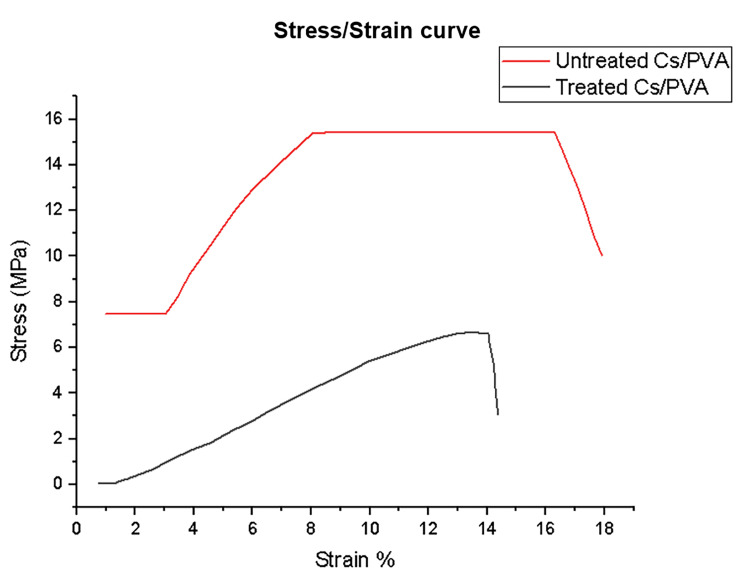
Stress/strain curves of treated and untreated chitosan/polyvinyl alcohol nanofibrous scaffolds. Cs/PVA: chitosan/polyvinyl alcohol

The ultimate tensile strength of the heat-treated scaffolds was 6.67 ± 0.7 MPa compared to 8.54 ± 1.2 MPa of the untreated group, while the elongation at break decreased from 5.21 ± 1.1 mm to 1.97 ± 1.3 mm. The Young modulus was 35.8 ± 2.7 compared to 44.7 ± 3.5 for the other group, as shown in Figure 6.

Physical analysis

Contact Angle, Swelling Ratio, and Degradation Rate of CS/PVA NFS

**Figure 7:**
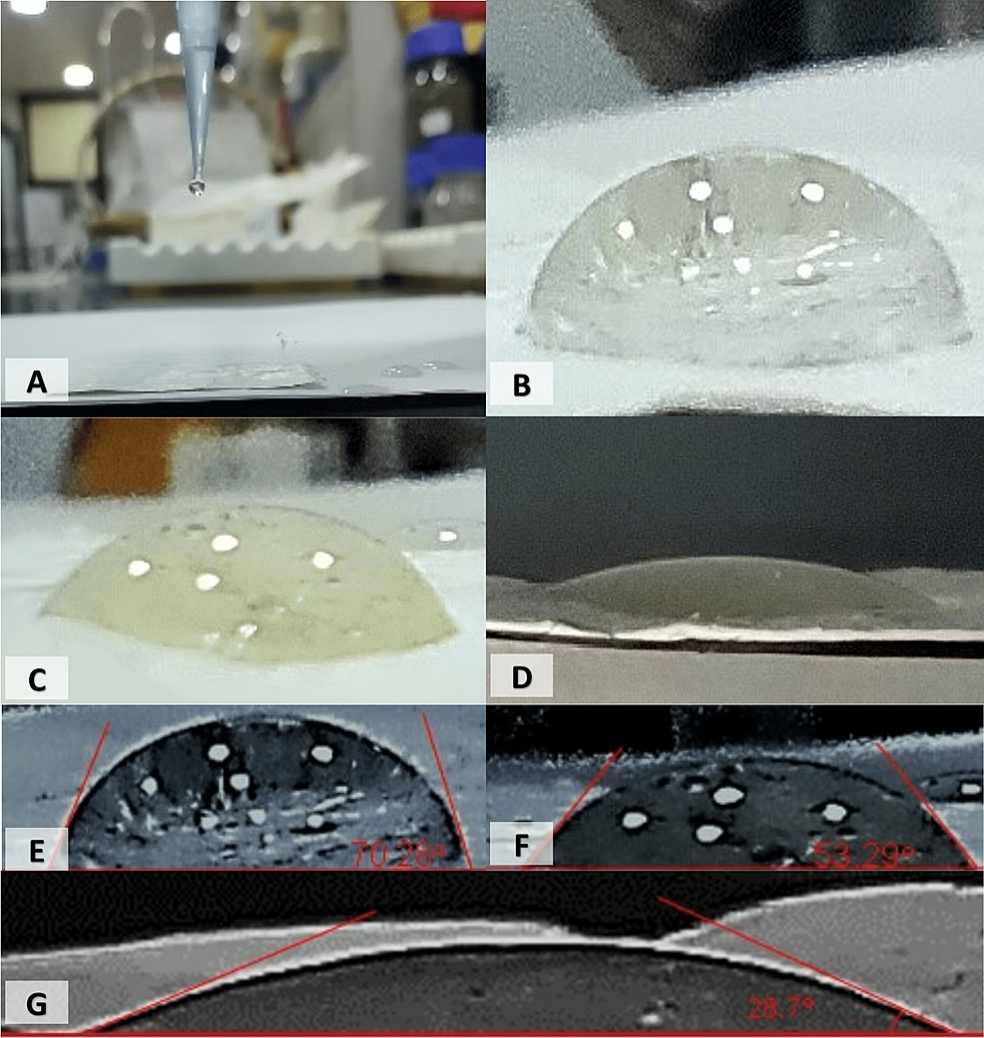
Photographic images of the water droplets on a surface and the calculation of contact angles of the chitosan/polyvinyl alcohol nanofibrous scaffolds. (A) The instrument used for water dropping. (B) At one second. (C) At 30 seconds. (D) At 60 seconds. (E) Contact angle measured at one second. (F) Contact angle measured at 30 seconds. (G) Contact angle measured at 60 seconds.

The mean contact angle of CS/PVA NFS immediately after drop stabilization was 72.3° ± 1.9°, and the angle decreased to 54.3° ± 0.7° and 29.5° ± 0.7° after 30 and 60 seconds, respectively, as shown in Figure 7 and Table 2.

**Table 2:** The raw data of the contact angles of the three samples of chitosan/polyvinyl alcohol nanofibrous scaffolds along with the mean values and standard deviations.

Samples	1 second	30 seconds	60 seconds
First sample	70.3°	53.3°	28.7°
Second sample	75.1°	55.4°	30.6°
Third sample	71.5°	54.3°	29.2°
Mean	72.3°	54.3°	29.5°
Standard deviation	1.9°	0.7°	0.7°

**Figure 8:**
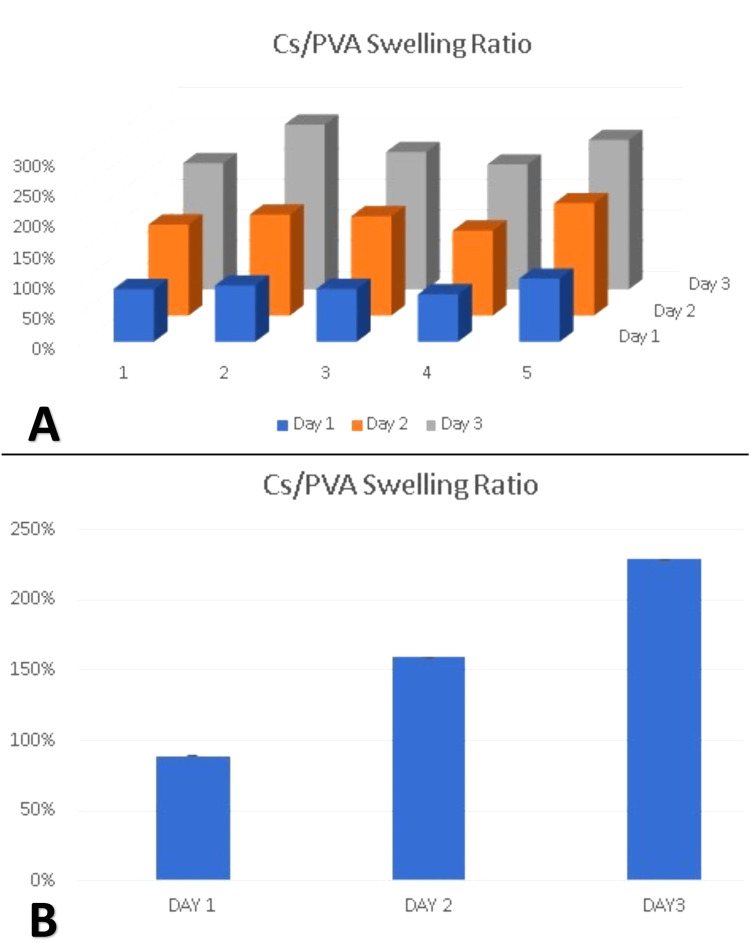
Swelling ratio of the chitosan/polyvinyl alcohol nanofibrous scaffolds within three days. (A) Assessment of swelling ratio in each of the five samples. (B) The average values of the five samples on each assessment day. Cs/PVA: chitosan/polyvinyl alcohol

The mean swelling ratio of CS/PVA NFS was 89% ± 6.8 on the first day, and the ratio increased on the second and third day to 159.4% ± 12.72 and 225.4% ± 17.44, respectively (Figure 8). No further swelling occurred in the following days.

**Figure 9:**
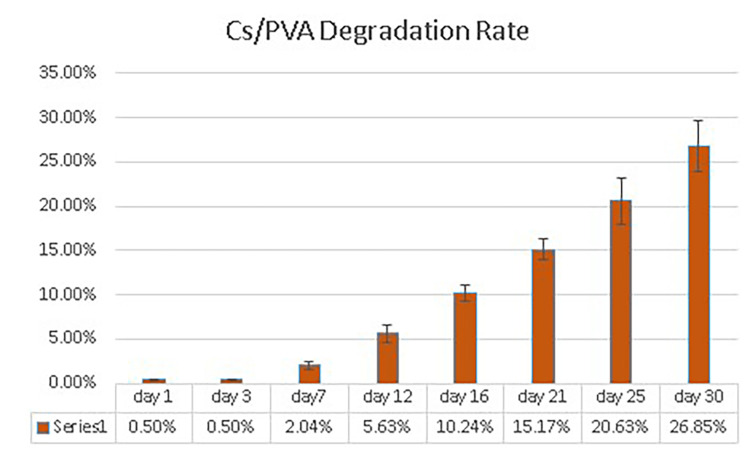
Degradation over time of the chitosan/polyvinyl alcohol nanofibrous scaffolds. Cs/PVA: chitosan/polyvinyl alcohol

The mean degradation rate of the CS/PVA NFS in PBS was 0.5% ± 0.02 on the first day. The degradation rate increased gradually over time to 2.04% ± 0.41, 10.24% ± 0.85, and 26.85% ± 2.89 on the 7th, 16th, and 30th days, respectively, as shown in Figure 9 and Table 3.

**Table 3:** The degradation values (mean percentage of weight loss) of the chitosan/polyvinyl alcohol nanofibrous scaffolds over 30 days of observation.

Days	Mean percentage of weight loss	Standard deviation
Day 1	0.50%	0.02
Day 3	0.50%	0.04
Day 7	2.04%	0.41
Day 12	5.63%	0.97
Day 16	10.24%	0.85
Day 21	15.17%	1.25
Day 25	20.63%	2.62
Day 30	26.85%	2.89

Biological analysis

Protein Adsorption and Cell Viability

The results of the in vitro protein adsorption test showed that 60% of the protein was adsorbed onto the CS/PVA NFS on the first and second days, and a 54.7% adsorption ratio was observed on the third day, as shown in Table 4.

**Table 4:** Protein adsorption profile of chitosan/polyvinyl alcohol nanofibrous scaffolds over three days of observation.

Time	Initial protein concentration	Free protein (mg/mL)	Adsorbed protein (mg/mL)	Adsorption (%)
Day 1	10 mg/mL	4	6 ± 2.0	60%
Day 2		4.01	5.99 ± 0.14	60%
Day 3		4.53	5.47 ± 2.35	54.7%

**Figure 10:**
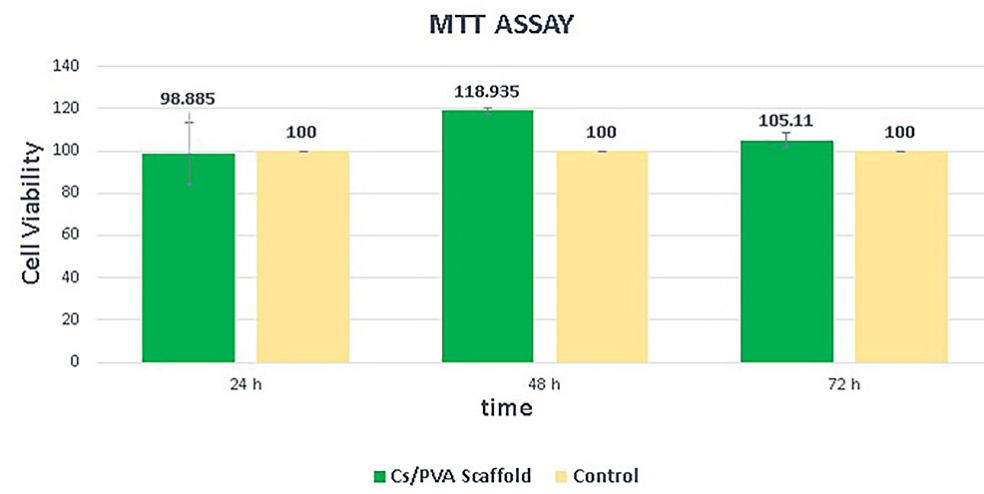
Cell viability of human adipose tissue-derived mesenchymal stem cells over 72 hours of observation. MTT: 3-(4,5-dimethylthiazol-2-yl)-2,5-diphenyltetrazolium bromide

**Figure 11:**
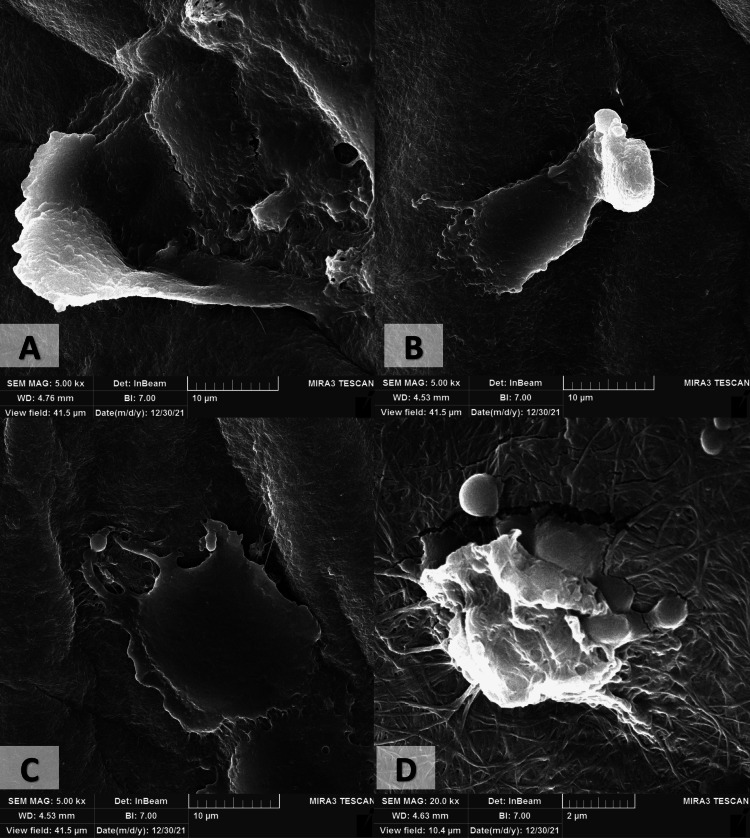
Field emission-scanning electron microscope images for the cultured human adipose tissue-derived mesenchymal stem cells on the chitosan/polyvinyl alcohol nanofibrous scaffolds. (A-C) At 5,000× magnification. (D) At 20,000× magnification.

The proportion of cell viability of the hAD-MSCs on the CS/PVA NFS through the MTT assay was 98.89% ± 14.67 on the first day, 118.94% ± 1.08 on the second day, and 105.11% ± 3.64 on the third day, as shown in Figure 10. Moreover, cells were successfully attached and proliferated on these scaffolds, as shown in Figure 11.

## Discussion

To confirm the existence of both chitosan and PVA, the FTIR was carried out. The FTIR analysis revealed that chitosan and PVA were homogenously blended and successfully electrospun together, as evident by the presence of characteristic peaks of both polymers, and the shift of the peaks and/or the change in the intensity of their absorption peaks after electrospinning and heat treatment were due to increase in hydrogen bonding between chitosan and PVA [41].

One of the advantages of using electrospinning to produce nanofibers is the close similarity of these fibers to the fibers of ECM concerning shape, diameter, orientation, and porosity. Moreover, the nano-sized diameter of the produced fibers led to an increased surface area-to-volume ratio. These factors would enhance the biological and cellular responses [13]. The randomly oriented smooth nanofibers observed by the FE-SEM micrographs with their average diameter of 172.7 ± 56.8 nm and pore size of 0.54 ± 0.17 µm are similar to those of ECM along with the increase in surface area, at least theoretically supporting protein adsorption and cell adhesion, proliferation, and differentiation.

Porosity can be defined as the volume occupied by voids of the total volume of the nanofibrous scaffold and can be obtained using the fluid displacement method; ethanol was chosen as a displacing solution instead of water as the latter caused swelling of the fibers. The optimal porosity for cell penetration is 60-90% [42].

The porosity of the fabricated scaffolds was within this range (74.8% ± 3.3); the randomly oriented fibers, along with layered deposition of the fibers during electrospinning, created interconnected pores which would help cells to communicate with each other and create channels and passages for the new blood vessels and oxygen, nutrient, and exudate exchange [42].

In this study, the tensile property of CS/PVA NFS decreased with heat treatment. The decrease in mechanical properties in the group subjected to heat treatment was due to loss of water and increased crystallinity, thus decreasing the elasticity of the fibers [43]. In general, the overall decrease in the mechanical properties of CS/PVA NFS is due to the increased content of chitosan and randomly oriented fibers [38]. Nonetheless, these scaffolds can be used in non-load-bearing areas.

Contact angle tests can be used to evaluate the surface wettability and hydrophilicity of nanofibrous mats, which are crucial for protein adsorption and enhanced cell attachment, proliferation, and differentiation within the matrices of synthesized biomaterials. Scaffolds that possess high surface energy have better wettability and hydrophilicity than those with lower surface energy. In general, a surface with a WCA of more than 90° is considered hydrophobic, while those with WCA lower than 90° are hydrophilic [27]. Moreover, cell attachment on the mats is highly consistent with higher surface energy and WCAs of less than 75° [44]. In our study, CS/PVA scaffolds show a great hydrophilic nature and good wettability over time. The initial contact angle was 72.3° ± 1.9° immediately after drop stabilization, which decreased to 54.3° ± 0.7° and 29.5° ± 0.7° after 30 and 60 seconds, respectively. This could be attributed to the presence of amino and hydroxyl groups on the CS/PVA scaffold’s surface.

Studying the swelling behavior of biomedical scaffolds is important as it indicates their structural stability in an aqueous environment and their capability to allow cells, body fluids, and proteins to penetrate them [36]. The swelling ratio of CS/PVA nanofibrous mats increased over time from 89% ± 6.8 on the first day to 229% ± 21.68 on the third day and became stable after that. This could be attributed to the hydrophilic nature of the scaffold, as indicated by the contact angle, as well as to the higher porosity that led to an increased swelling ratio [39].

The in-vitro degradation rate of the heat-treated CS/PVA scaffolds was investigated in PBS for 30 days. Weight loss increased with time, and the scaffolds lost 26.9% ± 2.9 of their original weight by 30 days; the increased chitosan content could explain this in this scaffold and increased hydrogen bonding between chitosan and PVA during thermal treatment [41].

Protein adsorption onto fabricated nanofibrous scaffolds is key to improving cell adhesion and attachment and regulating cellular response [39]. The results of the in-vitro protein adsorption study on the fabricated CS/PVA scaffolds on days one, two, and three were discussed in the Results section. From this study, it can be observed that protein was adsorbed successfully onto the scaffolds and achieved equilibrium, and all attachment sites were filled quickly from the first day. This could be attributed to the higher positively charged chitosan content in our scaffold, which could bind more negatively charged proteins, the increased wettability, and the nanoporous nature of the CS/PVA scaffolds that led to the increased surface area [40].

A cell viability study is crucial for any scaffold to be applied in tissue engineering [45]. The MTT assay performed to determine the proliferation rate of AD-MSC on the CS/PVA NFS showed a steady increase in cell viability over time which means that CS/PVA scaffolds supported cell attachment and proliferation, and no signs of cytotoxicity were observed. The morphology of the AD-MSC on the CS/PVA scaffold was observed on day three by FE-SEM. Cells spread well on the scaffold’s surface with a homogenous, flat pattern and cytoplasmic extensions (filopodia) of cells interacting with the scaffold. It is worth noting that the fabricated scaffolds retained their architecture within the culture media during the experiment.

Higher magnification of the AD-MSC morphology provides a clear view of the importance to fabricate the scaffolds in a manner that resembles the ECM nanostructure of the tissues as it provides micropores, interlacing fibers, and rough sites for the cells to implant and extends their extensions to attach and interact with the surroundings. This could be attributed to the nanofibrous architecture that mimics the ECM and provides rough sites for cell adhesion, fiber’s diameter, increased porosity, and appropriate pore size, which provide mechanical interlocking, cell-to-cell communication, and the high surface area along with the biocompatibility, hydrophilic properties, and non-toxicity of the chitosan and PVA, which all favor cell implantation, attachment, proliferation, and migration [12,45].

Our findings were in line with many researchers who used at least chitosan in their research and different cells such as mouse osteoblast cells (MC3T3E1) [45] and L-929 fibroblast cells [12]. On the other hand, Agrawal and Pramanik reported in their in-vitro biocompatibility study that mesenchymal stem cells did not attach to the fabricated PVA/CS scaffolds and chitosan/silk fibroin/polyethylene oxide scaffolds and attributed that to inadequate growth factors within the culture media and/or to remaining acetic acid within the scaffolds which turned the formulation more acidic and unsupportive for cell growth and proliferation [38].

Limitations of the study

PVA is readily soluble in water and needs to be stabilized. In this study, heat treatment was used as an eco-friendly method. The current analysis did not evaluate the effect of different temperatures and treatment times to achieve the optimum conditions for scaffold stabilization. In addition, this work did not include a thermogravimetric analysis which would have given us detailed information regarding the physical and chemical behavior of the scaffolds during the thermal change over time. The degradability of CS/PVA NFS was tested in PBS only (pH = 7.4) and not in acidic and alkaline media.

## Conclusions

This study showed the possibility of preparing CS/PVA NFS by electrospinning. Chitosan was successfully blended and incorporated into the nanofibers. The scaffolds proved stable in an aqueous medium by thermal treatment with good wettability. Reasonable swelling ratios, degradation rates, and protein adsorption were obtained. Cultured cells attached and proliferated well on the nanofibrous mat with no signs of cytotoxicity, along with a good capability to adsorb proteins. The potential of these scaffolds to be used in tissue engineering applications in the oral cavity is evident.
